# Neoadjuvant stereotactic radiosurgery for brain metastases: Current evidence and clinical perspectives

**DOI:** 10.17305/bb.2025.12890

**Published:** 2025-09-03

**Authors:** Aybala Nur Ucgul, Ahmet Oguz Tugcu, Ozge Petek Erpolat

**Affiliations:** 1 Department of Radiation Oncology, Gulhane Training and Research Hospital, Ankara, Türkiye; 2 Department of Radiation Oncology, Faculty of Medicine, Gazi University, Ankara, Türkiye

**Keywords:** Brain metastasis, stereotactic radiosurgery, SRS, neoadjuvant treatment

## Abstract

Neoadjuvant stereotactic radiosurgery (SRS) has emerged as a promising strategy for managing brain metastases, offering several advantages over traditional postoperative approaches. By delivering targeted radiation prior to surgical resection, neoadjuvant SRS aims to enhance local tumor control, reduce the risk of leptomeningeal dissemination, and optimize treatment efficiency. Recent findings suggest that neoadjuvant SRS provides comparable, if not superior, local control compared to postoperative SRS, while exhibiting lower rates of radiation necrosis and leptomeningeal disease. However, uncertainties persist regarding optimal dosing regimens, treatment timing, and patient selection criteria, as factors such as tumor size, volume, and histology may significantly influence clinical outcomes. Additionally, while neoadjuvant SRS addresses challenges related to target delineation and delays associated with postoperative treatment, its long-term efficacy and integration with systemic therapies require further investigation. This review consolidates evidence from recent retrospective and prospective studies, focusing on key outcomes such as local control rates, radiation toxicity profiles, and overall survival.

## Introduction

Brain metastasis affects approximately 20% of cancer patients and represents a significant cause of morbidity and mortality in this population [1]. The prevalence of brain metastasis at the time of diagnosis or after diagnosis varies depending on the primary cancer type. For instance, brain metastases are present in 25% of patients with metastatic melanoma at the time of diagnosis, whereas only 2% of patients with gastrointestinal cancers exhibit brain involvement [2]. The cancers most likely to develop brain metastases after diagnosis include lung cancer, breast cancer, renal cell cancer, and melanoma [3, 4].

The management of brain metastases requires a multidisciplinary approach, incorporating surgery, radiotherapy, and systemic treatments [3, 5]. In patients with limited brain metastases or larger tumors causing significant mass effects, surgical resection is generally recommended [3]. However, despite surgical intervention, local recurrence rates remain as high as 50% [6]. To mitigate this risk, postoperative radiotherapy has been widely adopted and shown to reduce local recurrence rates in multiple studies [6, 7]. Historically, postoperative whole-brain radiotherapy (WBRT) was the standard approach. However, concerns regarding long-term neurotoxicity and cognitive decline have led to an increasing preference for postoperative stereotactic radiosurgery (SRS) as an alternative [8–10].

Radionecrosis (RN) after SRS is generally reported in less than 20% of treated lesions [11]. Factors contributing to the development of RN after SRS include radiation dose, treated volume, and the volume of brain tissue receiving a specific dose [12]. In postoperative SRS (post-SRS), the irradiated volume is larger due to the inclusion of the surgical cavity, which has been linked to an increased risk of RN [12, 13].

Additionally, post-SRS carries a high risk of leptomeningeal disease (LMD) due to tumor spillage during resection. The risk of LMD after post-SRS has been reported to range between approximately 10% and 20%, and in some series, up to 30% [14, 15]. It is established that LMD is an important cause of neurological death [16].

Another drawback of post-SRS is treatment compliance, as some patients may experience prolonged postoperative recovery, delaying the initiation of SRS. Such delays could negatively impact local tumor control [17].

The limitations of post-SRS have prompted research into the potential benefits of preoperative SRS (pre-SRS). The aim of this review is to evaluate the effectiveness and complications of neoadjuvant SRS for brain metastases.

### Literature search methods

A literature search was conducted in the PubMed, Embase, Scopus, and Cochrane Library databases. The search was limited to articles published in English up to January 1, 2025. The following keywords and their combinations were utilized: “brain metastases”, “neoadjuvant”, “preoperative”, “stereotactic radiosurgery”, and “radiosurgery”. The present study incorporated original research (retrospective or prospective) that evaluated the efficacy of preoperative SRS in patients with brain metastases, comparing it with postoperative or alternative management strategies.

### Neoadjuvant SRS

Pre-SRS is an alternative approach to delivering SRS to intact brain metastases before resection in patients with brain metastases. Pre-SRS has several advantages over post-SRS. Firstly, in the preoperative approach, the target volume and the volume of normal brain tissue receiving a dose are lower than in the postoperative approach, which could reduce the risks of complications of SRS, including RN. Secondly, the preoperative approach involves the irradiation of tumor cells before surgery, which results in a reduced likelihood of tumor spillage and LMD. Thirdly, given the absence of surgical complications, pre-SRS may facilitate improved patient compliance compared to postoperative approaches [18]. Finally, because of decreased oxygenation in the postoperative environment, pre-SRS can be more efficacious [19].

Early single-arm retrospective case series suggested that pre-SRS can decrease LMD and RN without a reduction in local control [18, 20–23]. One of the first series determined that the 6-month local control was 97.8% and the 2-year local control was 71.8% with pre-SRS, while LMD and RN were not observed in any patients [20]. Prabhu et al. [18] conducted the largest multi-center retrospective study involving 242 patients, reporting a 1-year local control rate of 85%. Meanwhile, the rates for LMD and RN were notably low at 6.1% and 7.4%, respectively. Furthermore, three recently published prospective trials have demonstrated that the 1-year local control rate is over 75%, while the rates of RN and LMD are less than 10% with neoadjuvant single-fraction SRS [24–26].

### Neoadjuvant vs adjuvant SRS

Current guidelines recommend post-SRS to reduce local failure, with previous studies demonstrating local control rates ranging from 60.5% to 91% with this approach [3, 11]. However, it is recognized that post-SRS is associated with a high rate of LMD and RN, with 14% and 19% rates, respectively [11]. Consequently, in addition to single-arm trials investigating pre-SRS, several studies compared the effectiveness and safety of pre- and post-SRS in patients with brain metastases. Patel et al. first compared pre-SRS and post-SRS in brain metastases. The findings indicated that LMD and RN were significantly lower in the pre-SRS arm compared to the post-SRS arm (2-year LMD: 3.2% vs 16.6%, *P* ═ 0.01; 2-year RN: 4.9% vs 16.4%, *P* ═ 0.01). However, local control (*P* ═ 0.24) and overall survival (*P* ═ 0.1) were found to be non-significantly different [14]. Subsequently, Patel et al. compared pre-SRS with postoperative WBRT, hypothesizing that WBRT would be associated with a reduced incidence of LMD and RN. The study found that the two groups had comparable local control, overall survival, and LMD rates. However, the rate of RN was found to be higher in the pre-SRS arm than in the postoperative WBRT arm (5.6% vs 0%). It should be noted that the impact on cognitive function was not investigated in this trial [27]. Thus, the authors concluded that pre-SRS could be considered an alternative to postoperative SRS/WBRT [14, 27].

A previous meta-analysis compared single-arm studies evaluating pre-SRS and post-SRS. The analysis found that local recurrence was lower in pre-SRS trials (11%) compared to post-SRS trials (17.5%). However, this difference did not translate into improved overall survival rates. The authors attributed this to the relatively low incidences of LMD and deaths from brain metastasis or systemic progression. Additionally, the incidence of RN was found to be comparable between the two groups. However, a significantly higher rate of LMD was observed in the post-SRS trials (12.3%) compared to the pre-SRS trials (4.4%). It is important to note that this meta-analysis had certain limitations. Firstly, the number of pre-SRS trials included was considerably lower than that of post-SRS trials, with 517 pre-SRS patients from 6 trials and 3,129 post-SRS patients from 33 trials. Secondly, all studies were single-arm designs, and the duration of follow-up varied significantly between them. Finally, local recurrence, LMD, and RN definitions varied across the studies [28].

A more homogenous meta-analysis comparing pre-SRS with post-SRS included four trials conducted between 2015 and 2024. The findings indicated that both treatment plans were comparable in terms of overall survival, with a hazard ratio (HR) of 0.79 (95% confidence interval [CI]: 0.62–1.02, *P* ═ 0.07), and LF-free survival, with an HR of 1.38 (95% CI: 0.79–2.40, *P* ═ 0.26). Additionally, the 1-year risk of RN and LMD was significantly lower in the preoperative group, with *P* values of 0.02 for RN and 0.03 for LMD. Unlike the previously mentioned trial, this meta-analysis focused on trials that ensured similar criteria were used to measure outcomes and adverse events [29].

Recent translational and clinical studies suggest that pre-SRS not only provides local control but also modulates immune responses, with evidence from both tumor-level analyses [30] and systemic immune profiling after stereotactic radiotherapy [31]. Emerging molecular insights into radioresistance, such as NRF2-mediated pathways [32], may inform future strategies to optimize treatment outcomes.

**Figure 1.**
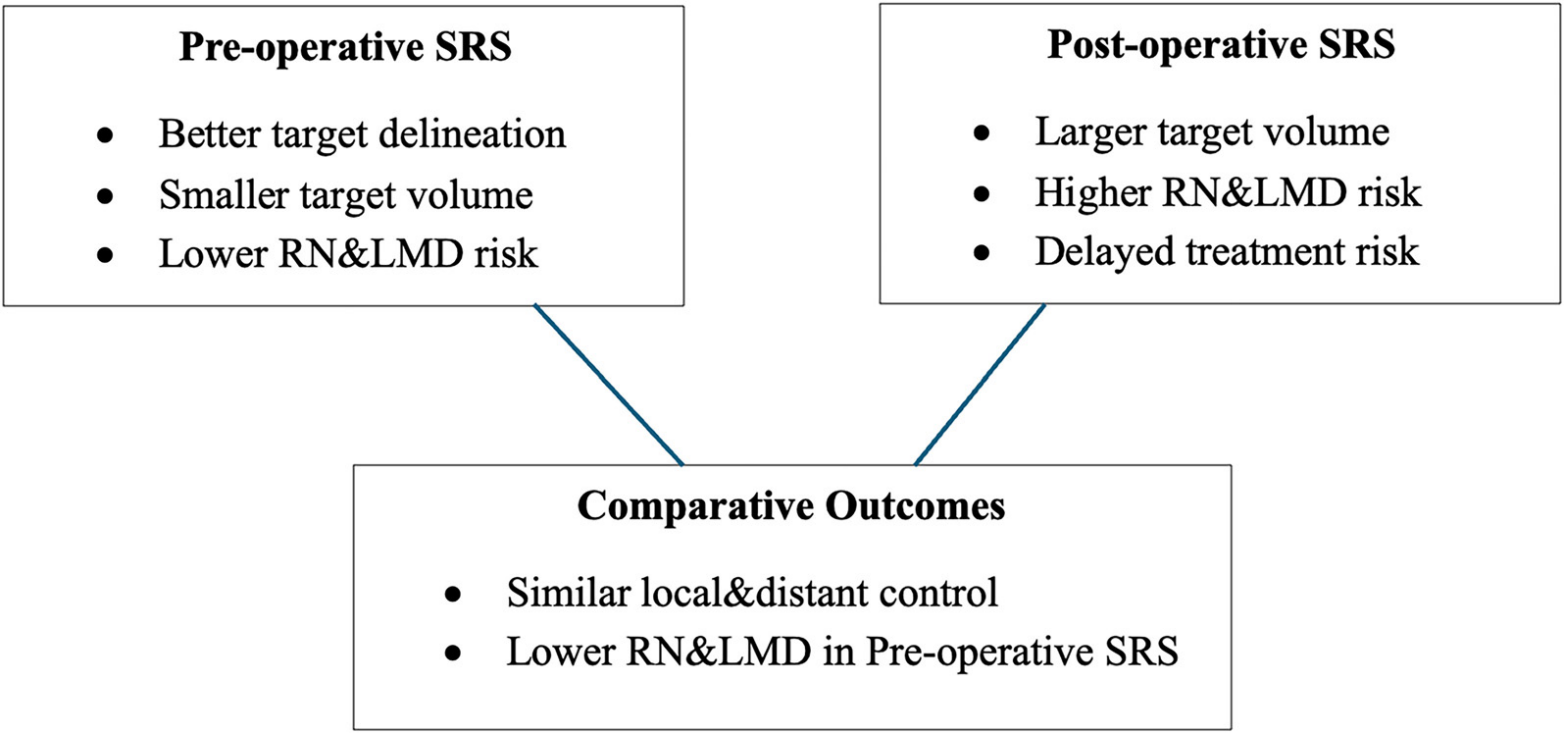
**Schematic comparison of pre- and post-SRS for brain metastases.** Pre-SRS allows for better target delineation, smaller treatment volumes, and a lower risk of RN and LMD. Post-SRS generally involves larger target volumes, carries a higher risk of RN and LMD, and may be associated with treatment delays. Comparative outcomes from the meta-analysis indicate similar local and distant control, with a lower risk of RN and LMD in the preoperative group. Abbreviations: SRS: Stereotactic radiosurgery; RN: Radiation necrosis; LMD: Leptomeningeal disease.

**Figure 2.**
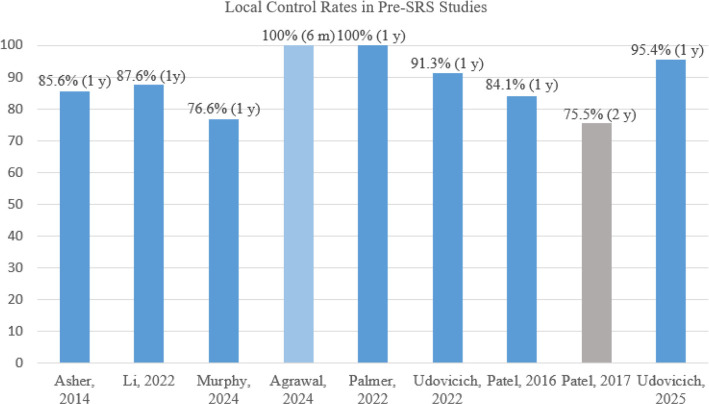
**Reported local control rates in preoperative SRS studies for brain metastases.** Abbreviation: SRS: Stereotactic radiosurgery.

**Figure 3.**
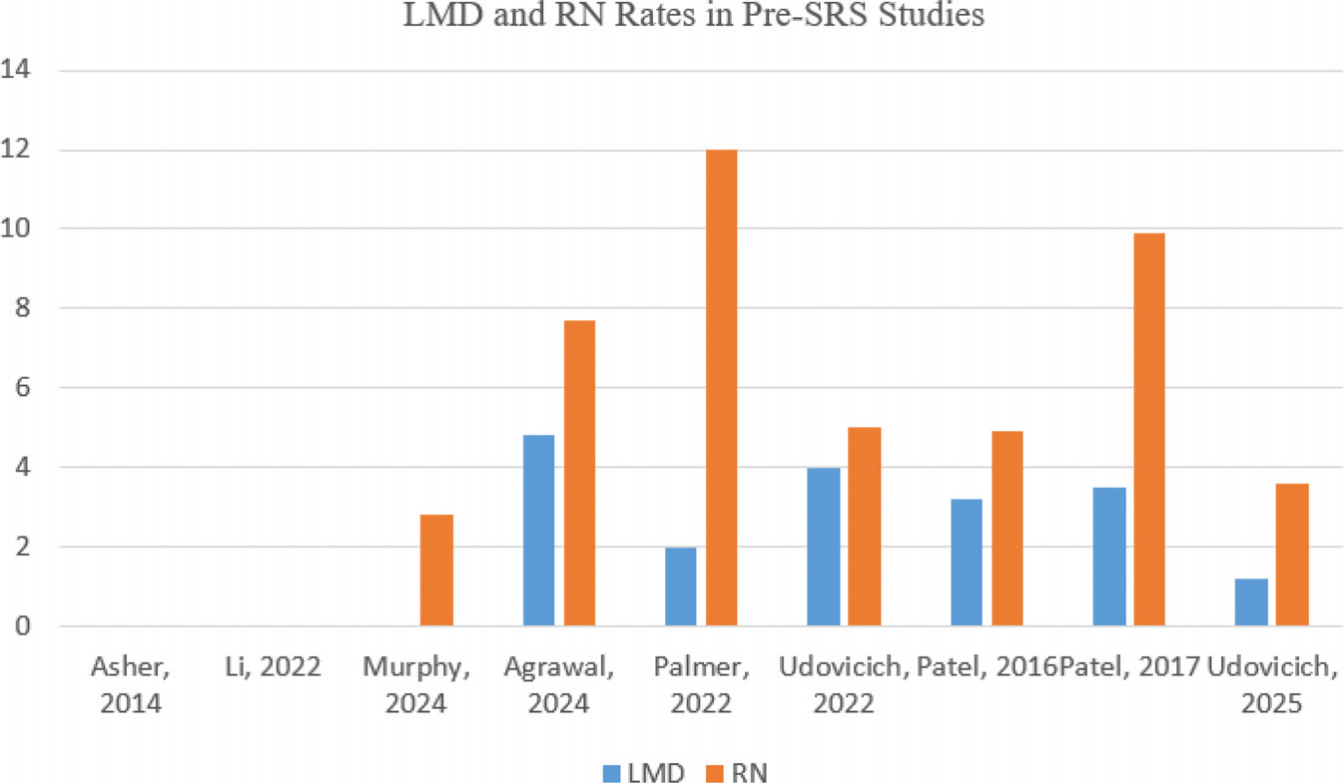
**Reported incidence of radionecrosis (RN) and leptomeningeal disease (LMD) in preoperative SRS studies for brain metastases.** Abbreviations: SRS: Stereotactic radiosurgery; RN: Radiation necrosis; LMD: Leptomeningeal disease.

**Table 1:** A summary of clinical trials assessing the efficacy of neoadjuvant SRS

**Author**	**Trial design**	**Groups**	**Number of patients**	**Median dose (range)**	**Median volume (range)**	**LC (timepoint)**	**OS (timepoint)**	**LMD**	**RN**
Asher, 2014 [21]	Prospective	Pre-SRS	47	14 Gy (11.6–18 Gy)	8.49 cc (0.89–46.7 cc)	85.6% (1 year)	60% (1 year)	0%	0%
Li, 2022 [22]	Retrospective	Pre-SRS	24	17 Gy (14–21 Gy)	10.1 cc (1.8–14.9 cc)	87.6% (1 year)	70% (1 year)	0%	NR
Murphy, 2024 [25]	Prospective	Pre-SRS	35	18 Gy for 2–3 cm 15 Gy for 3–4 cm 12 Gy for >4 cm	18.1 cc (4.4–64.8 cc)	76.6% (1 year)	59% (1 year)	0%	2.8%
Agrawal, 2024 [26]	Prospective	Pre-SRS	47	RTOG9005 dosing criteria	NR	100% (6-months)	17.6 months (Median)	4.8%	7.7%
Palmer, 2022 [23]	Retrospective	Pre-fSRT	53	24–25 Gy/3–5 fx	19 cc (12–28 cc)	100% (1 year)	70% (1 year)	2%	12%
Udovicich, 2022 [24]	Retrospective	Pre-fSRT	28	20–24 Gy/1–3 fx	4.5 cc (3.11–8.9 cc)	91.3% (1 year)	60.1% (1 year)	4%	5%
Patel, 2016 [29]	Retrospective	Pre-SRS Post-SRS	66 114	14.5 Gy 18 Gy	8.3 cc (0.89–46.8 cc) 9.24 cc (0.68–54.6)	84.1% (1 year) 87.4% (1 year)	NR	3.2% 16.6%	4.9% 16.4%
Patel, 2017 [30]	Retrospective	Pre-SRS Adj WBRT	66 36	30–37.5 Gy/10–15 fx	8.3 cc 15.3 cc	75.5% 74.9% (2 year)	59% (1 year) 55% (1 year)	3.5% 9%	9.9% 0%
Udovicich, 2025 [33]	Retrospective	Pre-SRS Pre-fSRT	100 89	18 Gy (16–20 Gy) 24–27.5 Gy/3 fx-5 fx	10.7 cc (5.6–18.9 cc)	95.4% (1 year)	66.3% (1 year)	1.2% (1 year)	3.6% (1 year)

Abbreviations: Pre-SRS: Neoadjuvant stereotactic radiotherapy; Pre-fSRT: Neoadjuvant fractionated stereotactic radiotherapy; Post-SRS: Adjuvant stereotactic radiotherapy; WBRT: Whole brain radiotherapy; Gy: Gray; fx: Fraction; NR: Not reached; LC: Local control; OS: Overall survival; LMD: Leptomeningeal disease; RN: Radionecrosis.

In summary, current meta-analyses show that pre-SRS is safe and effective. However, most pre- or post-SRS studies are retrospective and involve relatively small patient samples. Several ongoing clinical trials, including recent feasibility efforts [33], use more consistent inclusion criteria and more precise definitions of outcomes and adverse events to address these research gaps. The results of these studies will help establish the role of pre-SRS in managing brain metastases. A comparative summary of pre- and post-SRS approaches is presented in Figure 1. Preoperative SRS studies and their local control outcomes are summarized in Figure 2. Additionally, rates of radionecrosis and leptomeningeal disease are presented in Figure 3.

### Neoadjuvant SRS techniques

The contouring and planning of pre-SRS are generally more straightforward than those of post-SRS, primarily due to the absence of uncertainties regarding the surgical cavity. In pre-SRS, the gross tumor volume (GTV) is determined using contrast-enhanced T1-weighted magnetic resonance imaging (MRI), which should be conducted shortly before initiating SRS. The planning target volume (PTV) margin ranges from 0 to 2 mm, depending on the clinical immobilization technique [34, 35].

The optimal dose-fractionation scheme for preoperative radiotherapy remains unclear. Numerous studies have investigated the effectiveness of pre-SRS, which can be administered as either single-fraction SRS or fractionated stereotactic radiotherapy (fSRT). Most trials utilizing single-fraction SRS delivered doses between 14 and 20 Gy, while those employing fSRT administered doses ranging from 24 to 30 Gy, divided into 3–5 fractions. The characteristics of trials assessing pre-SRS/fSRT are outlined in Table 1. Although local control outcomes were comparable for both treatment schedules, there is a notion that higher biologically effective doses (BED) can be achieved with fractionated treatment, particularly for larger tumors [36]. The recent INTERNEO pooled individual-patient analysis reported that while multifraction pre-SRS schedules were associated with a significantly higher risk of RN (HR 5.85, *P* ═ 0.02), they did not confer an improvement in local control compared with single-fraction treatments [37].

It is important to acknowledge the limitations of this review when interpreting the findings. First, the included studies display substantial heterogeneity in outcome definitions. For instance, RN was variably reported as radiographic changes alone or as symptomatic cases requiring clinical management, while LMD was diagnosed using differing criteria such as imaging findings or cerebrospinal fluid cytology. These inconsistencies complicate cross-study comparisons and interpretation of results. Secondly, the extant evidence base is predominantly composed of retrospective case series, with only a few randomized trials. This overreliance on lower-level evidence reduces the overall confidence in effect estimates. It also underscores the need for prospective, standardized studies to better define the comparative benefits of pre-SRS vs post-SRS.

## Conclusion and clinical implications

Neoadjuvant SRS is an emerging option for managing brain metastasis. Current studies demonstrate that pre-SRS offers similar local control compared to post-SRS while reducing LMD and RN. The findings indicate that its use is appropriate in certain cases, particularly for patients with large, surgically resectable lesions, where the objective is to minimize postoperative complications. In light of current practices, close collaboration between neurosurgery and radiation oncology teams, as well as individualized treatment planning, remains imperative. Further randomized trials are needed to establish its role as a standard treatment.
